# Estimating the Fraction of Non-Coding RNAs in Mammalian Transcriptomes

**DOI:** 10.4137/bbi.s443

**Published:** 2008-03-01

**Authors:** Yurong Xin, Giulio Quarta, Hin Hark Gan, Tamar Schlick

**Affiliations:** 1 Department of Chemistry, 251 Mercer Street, New York University, New York, NY 10012, U.S.A; 2 Courant Institute of Mathematical Sciences, 251 Mercer Street, New York University, New York, NY 10012, U.S.A

**Keywords:** randomness test, fraction model, putative non-coding RNA

## Abstract

Recent studies of mammalian transcriptomes have identified numerous RNA transcripts that do not code for proteins; their identity, however, is largely unknown. Here we explore an approach based on sequence randomness patterns to discern different RNA classes. The relative *z*-score we use helps identify the known ncRNA class from the genome, intergene and intron classes. This leads us to a fractional ncRNA measure of putative ncRNA datasets which we model as a mixture of genuine ncRNAs and other transcripts derived from genomic, intergenic and intronic sequences. We use this model to analyze six representative datasets identified by the FANTOM3 project and two computational approaches based on comparative analysis (RNAz and EvoFold). Our analysis suggests fewer ncRNAs than estimated by DNA sequencing and comparative analysis, but the verity of our approach and its prediction requires more extensive experimental RNA data.

## Introduction

The rapid progress in large-scale screening of cellular transcriptional output (termed the transcriptome) has dramatically increased the repertoire of transcripts expressed in mammalian cells (Bertone et al. 2004; Carninci et al. 2003; Carninci et al. 2005; Cheng et al. 2005; Kampa et al. 2004; Kapranov et al. 2002; Numata et al. 2003; Ota et al. 2004). Full-length sequencing data (Carninci et al. 2005; Ota et al. 2004) indicate that about 50% of the transcripts are likely to be non-coding RNAs (ncRNAs). Known ncRNAs are involved in a variety of cellular functions, such as gene regulation, rRNA modification, splicing, RNA editing, mRNA degradation. The currently-identified ncRNAs are far less abundant than protein-coding RNAs in mammalian transcriptomes. The large number of putative ncRNAs, however, leads us to speculate that the number of ncRNAs will continue to grow. For those putative ncRNAs, although experimental techniques, such as Northern blot, microarray analysis, and RT-PCR, are providing expression data (Carninci et al. 2005; Cheng et al. 2005; Kampa et al. 2004; Ota et al. 2004; Ravasi et al. 2006), most biological functions remain unknown. Determining the identity of these putative ncRNAs is important: Do these transcripts correspond to genuine ncRNAs with biological functions, or to other RNAs that may be biological or experimental artifacts, non-functional transcripts, or transcriptional noise (Huttenhofer et al. 2005; Johnson et al. 2005; Soares and Valcarcel, 2006).

The FANTOM (Functional Annotation of Mouse) database, which aims to generate the transcriptional landscape of the mouse genome, has identified 34,030 manually annotated putative ncRNAs in the FANTOM3 release, among which 2886 sequences are annotated by the most stringent criteria (Carninci et al. 2005). Only a small population of the putative ncRNAs is known ncRNAs, such as miRNAs and snoRNAs, and others are unknown RNA transcripts.

Computational approaches such as RNAz (Washietl et al. 2005b; Washietl et al. 2005a) and EvoFold (Pedersen et al. 2006) have employed evolutionary conservation of secondary structures, thermodynamic stabilities, and phylogenetic sequence analysis to predict tens of thousands of conserved RNA secondary structural elements in the human genome. The largest dataset predicted by RNAz contains 91,676 RNA structures conserved at least in four mammals (human, mouse, rat, and dog) in non-coding regions (coding exons are removed). The EvoFold program predicts 48,479 RNA structures from various genomic locations (coding, UTR, intronic, and intergenic), of which 517 sequences are classified as ncRNA candidates by the program. However, less than 1% of the predictions by RNAz and EvoFold are known ncRNAs (Pedersen et al. 2006; Washietl et al. 2005a) and others are new RNA folds.

Despite various predictions concerning the biological functions of putative ncRNAs (Huttenhofer et al. 2005; Johnson et al. 2005; Mattick and Makunin I.V. 2006; Soares and Valcarcel, 2006), progress has been slow in identifying associated biological roles in cells. An exhaustive functional characterization of unknown RNA transcripts by experiments is prohibitive. However, determining the fraction of genuine non-coding RNAs in those putative ncRNAs from either experimental or computational data will advance our understanding of the composition of mammalian transcriptomes and the general importance of ncRNAs for cellular function. An approach we explore here is a systematic assessment based on statistical features for the known ncRNA sequences.

Because biological sequences are not purely random, statistical tests might help screen sequences of interest. Previous works on statistical properties of nucleotide sequences have been geared toward characterization of coding/non-coding regions (Almirantis, 1999; Herzel and Grosse, 1997; Kugiumtzis and Provata, 2004; Peng et al. 1992), sequence complexity (Abel and Trevors, 2005; Adami and Cerf, 2000), and evolutionary patterns (Dehnert et al. 2005b; Dehnert et al. 2005a). Moreover, it was found that ribosomal RNAs could be discriminated from random sequences by using statistical measures (Almirantis, 1999). Thus, quantifying sequence characteristics may reveal some aspects of functional features (such as general class type) based on sequence properties.

Here, we use relative *z*-scores of missing motifs to analyze characteristic global features for specific nucleotide sequence classes. We find that the relative *z*-score, derived from the monkey test (Marsaglia and Zaman, 1993) for assessing random number generators (RNGs), helps classify six nucleotide sequence classes into three clusters in decreasing degree of randomness: (1) genome/intergene/intron, (2) mRNA/ncRNA, and (3) repeat. We use this characteristic relative *z*-score of the ncRNA class to predict the ncRNA fraction in putative ncRNAs as determined in the FAN-TOM3 database and by computational programs RNAz and EvoFold.

## Materials and Methods

### Nucleotide sequence classes

We compare the degree of randomness of the following nucleotide sequence classes: genome, intergene, intron, mRNA (or coding sequence), and ncRNA. The genome, intergene, intron, and mRNA classes are generated from RefSeqs (Pruitt et al. 2005) in Supplementary Table S1. The ncRNA class contains 7,698 representative ncRNA genes from Noncode (Liu et al. 2005), RNAdb (Pang et al. 2005), Rfam (Griffiths-Jones et al. 2005), and European ribosomal RNA database (Wuyts et al. 2004) (Table 1). Noncode and RNAdb have low redundancy, while the remaining three sources have multiple copies. In order to determine the effect of multiple copies in randomness analysis, we create two other versions that have fewer or no rRNAs, tRNAs, or spliceosomal RNAs. We also create an additional class, the repeat sequence class, as a control: a long concatenated “sequence” is generated by repeating a 1,024-nt random sequence 2,048 times to form a 2,097,152-nt repetitive sequence.

To generate the fraction model, we use the ncRNA class and mouse genomic, intergenic, and intronic sequences (Supplementary Table S2). Mouse genomic and intergenic sequences have lengths of 10^8^ nt or more, so we cut them into 2000-nt segments which can conveniently be manipulated and shuffled.

### Putative ncRNA datasets

We analyze six putative ncRNA datasets identified by experimental and computational methods (Table 2). The two experimental datasets come from the FANTOM3 database of full-length cDNAs that do not code for proteins (Carninci et al. 2005). The computational datasets are formed by predictions from programs RNAz (Washietl et al. 2005b) and EvoFold (Pedersen et al. 2006). The sequences predicted by RNAz are grouped into three datasets based on different number of conserved organisms and p-values: (1) the set1.P0.5 dataset is predicted with sequences conserved at least in human, mouse, rat, and dog at p > 0.5; (2) the set1.P0.9 dataset is predicted at p > 0.9; (3) the set2.P0.5 dataset is predicted with sequences conserved at least in human, mouse, rat, dog, and chicken at p > 0.5. The sequences predicted by EvoFold are conserved in eight species (human, chimpanzee, mouse, rat, dog, chicken, pufferfish, and zebrafish).

### The randomness (monkey) test

The monkey test assesses sequence randomness based on a χ^2^ distribution of goodness-of-fit measure (∑(*OBS–EXP*)^2^/*EXP*) for the overlapping *k*- and *k-1*-letter words (Marsaglia, 2005; Marsaglia and Zaman, 1993). This test can assess both uniformity and independence of random sequences. We employ an applied version of the monkey test, the DNA test (Marsaglia and Zaman, 1993), in our study. In the DNA test, the number of missing *k*-letter words in a long sequence approximately follows a normal distribution whose mean and variance depend on the word and alphabet sizes. Thus, the *z*-score can quantify the degree of randomness:

(1) $$z=\frac{missing-mean}{std}$$

where the number of *missing* words is an observed value, *mean* is the theoretical average number of missing words, and *std* is the standard deviation.

**Figure 1:**
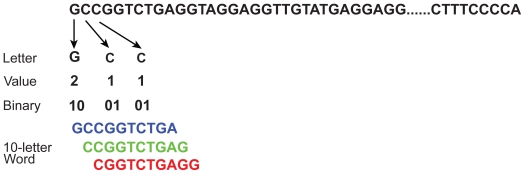
Application of the DNA monkey test to biological sequence analysis. The DNA test counts 10-letter missing words from a 4-letter alphabet (A, C, G, T/U). Three overlapping 10-letter words are shown in blue, green, and red, respectively.

In our analysis, we use the default parameters of the DNA test: it counts 10-letter words for a 2,097,152 letter sequence; the expected number of missing words behaves like a normal distribution with mean 141,909 and standard deviation 339. Figure 1 illustrates the conversion from nucleotide sequences to numeric sequences. Different word size and sequence length can also be applied to the DNA test.

### Applying the monkey test to DNA sequences

**Figure 2:**
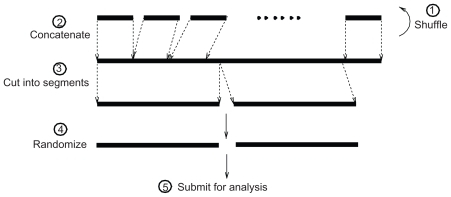
Sequence manipulation scheme for randomness analysis.

The DNA test requires long input sequences on the order of 10^6^ nt (2,097,152 nt) (Marsaglia and Zaman, 1993). We adopt the following procedure to analyze sequences whose lengths are shorter than 2,097,152 nt (Fig. 2): (1) we randomly shuffle original nucleotide sequences in a group by the Mersenne Twister RNG (Matsumoto and Nishimura, 1998); (2) we concatenate shuffled sequences into one sequence, our “concatenated sequence; “ (3) we cut that concatenated sequence into segments in the length of 2,097,152 nt; (4) we generate a random sequence with the same dinucleotide composition as the corresponding concatenated biological sequence; (5) we submit both biological and random sequences to the DNA test. Following this procedure, we generate at least 100 concatenated sequences and corresponding random sequences for a given sequence group and submit them to the DNA test. These pairs of *z*-scores result in relative *z*-scores which will be described in detail in Results. The distribution of the relative *z*-score of a given sequence group is summarized by mean, standard deviation, and range.

### Thermodynamic analysis

In addition to randomness test, we use a complementary thermodynamic analysis (the melting temperature and energy landscape analyses) to screen short putative ncRNAs in the FANTOM3 dataset. The melting temperature, defined by the peak of the heat capacity, is predicted by RNAheat (Hofacker et al. 1994; Mccaskill, 1990) from the Vienna RNA folding Package (Hofacker et al. 1994) (Version 1.6). The energy landscape, defined by the base pair dissimilarity “distance” between the minimum energy structure and each suboptimal structure, is measured by the “Valley Index” (Kitagawa et al. 2003). We use a 90% confidence ellipse in 2D plots of free energy vs. melting temperature and of free energy vs. Valley Index to determine stability of test structures as described in our previous work (Laserson et al. 2005). This method implies an error rate of 10%, i.e. random sequences pass the test in 10% of cases. The thermodynamic tests are applied to ten ncRNA families, namely tRNA, 5S rRNA, 5.8S rRNA, 6S RNA, SRP RNA, SL1 RNA, U6 RNA, UnaL2, snoRNA, and His3 in Rfam (Griffiths-Jones et al. 2005) and 151 putative ncRNAs (<400 nt) in FANTOM3 (Carninci et al. 2005).

## Results

### The relative *z*-score measures the degree of randomness

We employ the DNA test to generate a randomness measure, the *z*-score (Eq. 1) (Marsaglia and Zaman, 1993). Biological sequences often fail the DNA test because the number of missing motifs for biological sequences is often greater than the theoretical mean value (Eq. 1). Random sequences generated with nonuniform nucleotide compositions also fail the DNA test. Since dinucleotide composition has become standard to measure the background effect (Goni et al. 2004; Workman and Krogh, 1999), we use the random dinucleotide sequence as a control to the corresponding biological sequence. Thus, we define the relative *z*-score for the degree of randomness to be:

(2) $${\text{z}}_{\text{relative}}=\frac{{\text{z}}_{\text{bioseq}}}{{\text{z}}_{\text{di}-\text{random}}},$$

where *z*_bioseq_ is the *z*-score for a biological sequence and *z*_di-random_ is the *z*-score for the random dinucleotide control sequence. Higher-order background information (e.g. tri-nucleotide, tetra-nucleotide) can be considered in the future for model improvement. We also name the *z*-score (e.g. *z*_bioseq_ and *z*_di-random_) the absolute *z*-score to differentiate it from the relative *z*-score. A relative *z*-score close to 1 indicates that the dinucleotide composition is the major contributor to non-randomness; a value larger than 1 indicates that other sequence factors affect the non-randomness; a value near zero means that single-nucleotide composition rather than dinucleotide more likely causes the non-randomness. Below, we show that the relative *z*-score can distinguish various classes of biological sequences.

### The relative *z*-score classifies nucleotide sequence classes into three clusters

We then use the relative *z*-score to assess the degree of randomness of six nucleotide sequence classes: genome, intergene, intron, mRNA, ncRNA, and artificial repeat. The relative *z*-scores are obtained by running the DNA test on one hundred 2,097,152-nt concatenated sequences for each class from the three phylogenetic domains.

**Figure 3:**
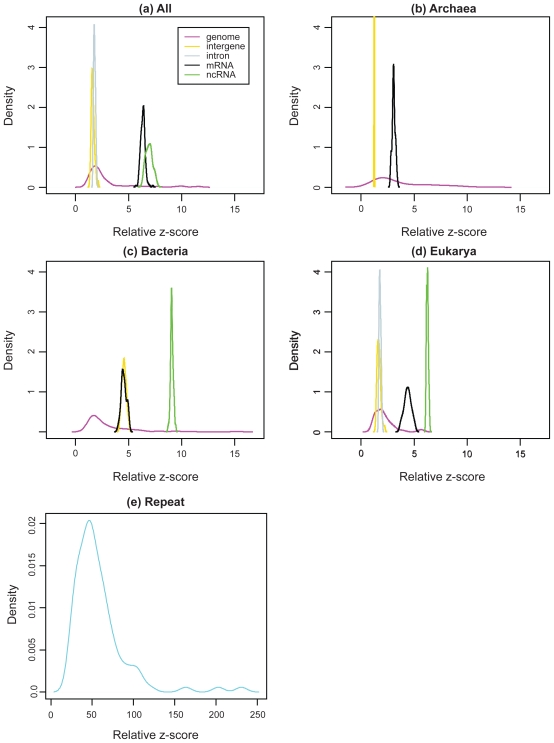
The degree of randomness (relative *z*-scores) for different sequence classes in the three phylogenetic domains by the DNA test: (**a**) the three-domain collection; (The intron class corresponds only to eukaryotes). (**b**) Archaeal dataset; (**c**) Bacterial dataset; (**d**) Eukaryotic dataset; (**e**) the repeat (control) dataset. The distribution of the relative *z*-score of each sequence class is estimated by the density function in R (R Development Core Team 2006). The color legend in the inset of (a) applies to (b), (c), and (d).

In the three-domain collection (Fig. 3a), the genome class displays the broadest distribution in sequence randomness (Table 3): 86% of relative *z*-scores fall into the region of 0.9–5.0. The inter-gene and intron classes have narrow relative *z*-score distributions overlapping with the peak of the genome distribution. The mRNA and ncRNA classes overlap with one another and have a lower degree of randomness than the previous three classes. The artificial repeat class spans a wide range and has the lowest degree of randomness among the six nucleotide sequence classes (Fig. 3e). As expected, regular motif patterns of the repeat class lead to a low degree of randomness. Thus, we find that the relative *z*-score partitions the six nucleotide sequences from the three domains into three clusters: (1) genome, intergene, and intron; (2) mRNA and ncRNA; and (3) repeat sequences.

After examining the randomness trends in all three domains, we analyze that in the three domains of life separately. In Eukarya, the six nucleotide sequence classes still form three clusters as they do in the three-domain collection (Fig. 3d). However, the genome/intergene/intron cluster has a narrower range than the one in the three-domain collection and the mRNA/ncRNA cluster is not as compact as that one in the three-domain collection. In Archaea, the genome, intergene, and mRNA classes form a cluster, with the mRNA class being less random than the intergene class (the intron and ncRNA classes are not available) (Fig. 3b). In Bacteria, the genome, intergene, and mRNA classes form a cluster, and the ncRNA class forms another one (Fig. 3c). The limited ncRNA dataset for bacteria, containing only a small number of available ncRNAs except for tRNAs and rRNAs, produces a relative *z*-score distribution mostly reflecting the randomness features of tRNA and rRNA families. Therefore, the clustering pattern among the six nucleotide sequence classes is maintained in Eukarya but changed in Archaea and Bacteria due to sequence bias in datasets. It also shows that the ncRNA class is less random than the other biological sequence classes in the three-domain collection and separate domains.

Overall, the relative *z*-score can partition two sequence clusters—genome/intergene/intron (more random) and mRNA/ncRNA—in the three-domain collection and Eukarya. For the ncRNA class, it is characterized by a distinct relative *z*-score in the three-domain collection, Bacteria and Eukarya. Its lower randomness than the genome/intergene/intron cluster may be explained by low motif diversity of the ncRNA class likely caused by the sequence conservation within some ncRNA families and RNA’s preference for specific recurrent motifs.

### Putative ncRNAs are not all functional

**Figure 4:**
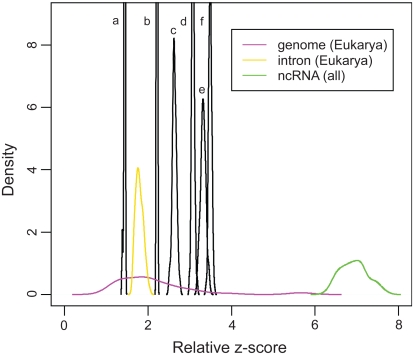
The degree of randomness of the six putative ncRNA datasets measured by the DNA test. The relative *z*-score distribution of the six datasets is denoted as follows: (a) EvoFold, (b) RNAz set2.P0.5, (c) FANTOM3 putative, (d) RNAz set1.P0.5, (e) FANTOM3 stringent and (f) RNAz set1.P0.9.

We now assess the six putative ncRNA datasets listed in Table 2 using the relative *z*-score. The total length of the EvoFold dataset is 1,869,205 nt which is shorter than the required length (2,097,152 nt) of the DNA test (Marsaglia and Zaman, 1993), so randomly selected ncRNAs are added to reach the length requirement. The relative *z*-score (1.437) of this “pseudo” EvoFold dataset is an estimate of the true value. Another “pseudo” EvoFold dataset created with additional genomic sequences has almost the same relative *z*-score (1.436). The DNA test result shows that none of the six datasets have a relative *z*-score close to the ncRNA class (Fig. 4). Instead, the six datasets form non-overlapping relative *z*-score distributions which fall in the genome/intergene/intron cluster. In order of decreasing degree of randomness, we have EvoFold, RNAz set2.P0.5, FANTOM3 putative, RNAz set1. P0.5, FANTOM3 stringent, and RNAz set1.P0.9.

Three explanations to the much smaller relative *z*-scores of these sequences compared to the ncRNA class are possible. First, these putative ncRNAs may consist of a mixture of real ncRNAs and other types of RNA transcripts (i.e. “RNA noise”). Second, while most of these putative ncRNAs may indeed have biological functions like known ncRNAs, the relative *z*-score is a poor indicator (e.g. ncRNAs are group I intron-like with a low relative *z*-score). Third, most of these putative ncRNAs may not have biological functions. Below, we develop a model to probe each possibility in turn.

### The fraction model for estimating the proportion of ncRNAs in test datasets

Whatever the fraction of true ncRNAs in these datasets, we attempt to formulate an approximate fraction model based on the assumption that the Fantom3 and computational datasets are mixtures of true ncRNAs and “RNA noise”. We thus construct a fraction model with various ncRNA to RNA noise ratios to match the relative *z*-scores of the test datasets.

**Figure S1:**
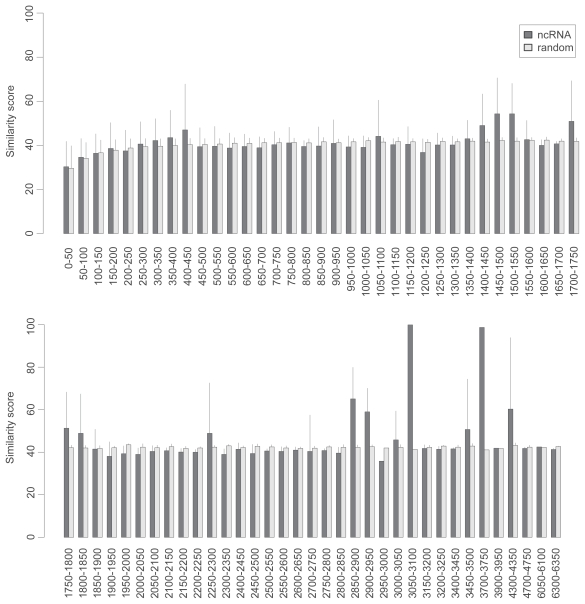
The pairwise sequence similarity of the ncRNA class. The ncRNA class is divided into subgroups with the window size of 50 nt. The sequence similarity within subgroups is analyzed by the EMBOSS program. The error bar shows the standard deviation of similarity scores in a subgroup.

**Figure S2:**
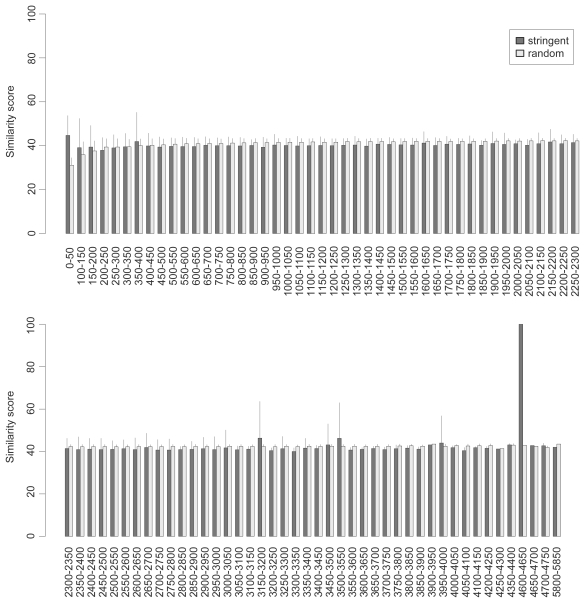
The pairwise sequence similarity of the FANTOM3 stringent dataset. The dataset is divided into subgroups with the window size of 50 nt. The sequence similarity within subgroups is analyzed by the EMBOSS program. The error bar shows the standard deviation of similarity scores in a subgroup.

**Figure S3:**
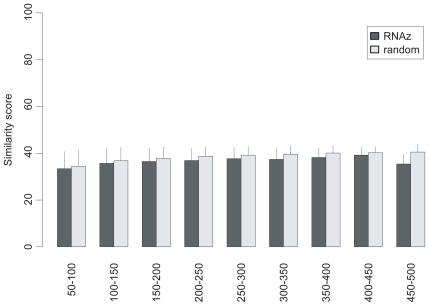
The pairwise sequence similarity of selected sequences of the RNAz dataset. The dataset is divided into subgroups with the window size of 50 nt. The sequence similarity within subgroups is analyzed by the EMBOSS program. The error bar shows the standard deviation of similarity scores in a subgroup.

**Figure S4:**
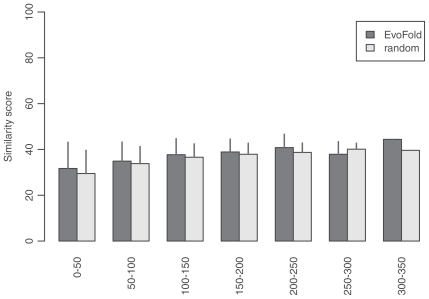
The pairwise sequence similarity of selected sequences of the EvoFold dataset. The dataset is divided into subgroups with the window size of 50 nt. The sequence similarity within subgroups is analyzed by the EMBOSS program. The error bar shows the standard deviation of similarity scores in a subgroup.

In our fraction model, the ncRNAs are sampled from the training ncRNA dataset defined in Methods which represents diverse RNA families in Noncode, RNAdb and several prominent families from Rfam and European ribosomal RNA database (Table 1). This choice of training dataset is reasonable because the Fantom3 dataset is highly heterogenous (broad length distribution, multiple experimental data sources (Carninci et al. 2005)) and the computational datasets represent whole genome scans. We thus assume the ncRNAs in the test datasets to be as diverse as those in the training dataset. Further analysis shows that the sequence properties of the training and test datasets are quite similar (Supplementary Figs. S1–S4). On the other hand, we may miss a part of new ncRNAs whose relative *z*-scores are different from the representative score of the training dataset.

To model RNA noise or background sequences, we use mouse intronic, intergenic, and genomic sequences. RNA noise may include biological and experimental artifacts, non-functional transcription, and transcriptional noise (Huttenhofer et al. 2005; Johnson et al. 2005; Soares and Valcarcel, 2006). Because our analysis shows that the putative ncRNAs fall in the cluster of genome/intergene/intron (Fig. 4), such RNA noise may come from genomic, intergenic, and/or intronic sources. Though RNA noise could also originate from mRNAs, this possibility is less likely because existing protein-coding sequences have been filtered (Carninci et al. 2005) or removed (Washietl et al. 2005a) a priori. Thus, we use mouse genomic, intergenic, and intronic sequences (Supplementary Table S2) to model RNA noise in mammalian transcriptomes under various ratios.

**Figure 5:**
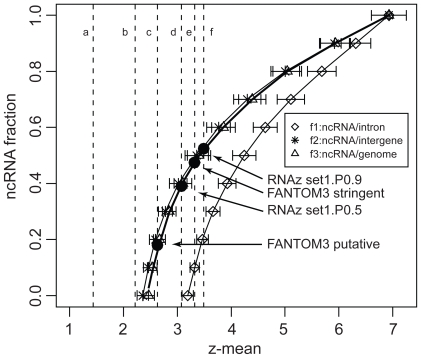
The model used to assess the ncRNA fraction in the FANTOM3, RNAz and EvoFold datasets. The mean relative *z*-score of the six datasets is shown in dashed lines in the same order as Fig. 4. Error bars show standard deviations of relative *z*-scores. The four predictions for the FANTOM3, the FANTOM3 stringent, the RNAz set1.P0.5 and the RNAz set1.P0.9 datasets by *f*_3_(*z*) are highlighted in bullets.

We consider three submodels based on the ncRNA/noise partitioning: (1) ncRNA/intron; (2) ncRNA/intergene; (3) ncRNA/genomic sequence. In each submodel, eleven ncRNA/noise ratios (0:10, 1:9, 2:8,…, 10:0) are applied to create concatenated sequences whose relative *z*-scores correspond to specific ncRNA fractions (from 0% to 100% with a resolution of 10%). The data points for the ncRNA fraction vs. the relative *z*-score defines a function *f*(*z*) where *z* is the mean of relative *z*-scores for a given ncRNA fraction. As expected, the three functions generated from the three submodels are monotonically increasing and converging to 1 as the fraction of ncRNAs increases (Fig. 5). The two functions *f*_2_(*z*) and *f*_3_(*z*) almost overlap with one another because the intergenic sequences cover about 68% of the genomic sequences (Supplementary Table S2). The ncRNA/intron submodel predicts lower ncRNA fractions than the other two submodels because the intron class has a larger average relative *z*-score than the genome and intergene classes.

Among the three submodels considered above, the third (ncRNA/genome) appears to simulate the putative ncRNA datasets most accurately. This expectation is supported by the following evidences: transcription of the mammalian genomes is wide (e.g. over 60% of the mouse genome is transcribed (Carninci et al. 2005)); and putative ncRNAs are transcribed from diverse genomic locations including coding regions, 5′- and 3′-UTRs, introns, and intergenic regions (Carninci et al. 2005; Cheng et al. 2005; Pedersen et al. 2006; Washietl et al. 2005a). Therefore, based on given relative *z*-scores of putative ncRNA datasets, we can use the fraction model (the ncRNA/genome submodel preferred) to predict the ncRNA fractions in mammalian transcriptomes.

In all three submodels, each ncRNA fraction has a relative *z*-score distribution represented by error bars which quantify the uncertainty of our prediction of ncRNA fractions (Fig. 5). At each data point, the error of *f* (*z*) is estimated by the standard deviation of *z*_mean_. Based on the eleven data points for each submodel, the average error of ncRNA fraction is about ±5%. Given this resolution of our model, we predict that less than 5% ncRNAs exist in a dataset with the mean relative *z*-scores below 2.

### Predicting the ncRNA fraction in the six representative datasets

We collect six putative ncRNA datasets from the three sources: the FANTOM3 database, and the predictions of RNAz and EvoFold. The overlap between one another is quite small: the RNAz program only found 781 conserved RNA folds out of more than 15,000 putative ncRNAs in the FAN-TOM2 database (Washietl et al. 2005a); about 6000 folds have at least partial overlaps between the predictions of RNAz and EvoFold.

We use the mean relative *z*-score of the two FANTOM3 putative ncRNA datasets to interpolate the ncRNA fraction with functions *f*_1–3_(*z*) (see Fig. 5 and Table 4). The relative *z*-score of the FANTOM3 dataset (dashed line c in Fig. 5) is out of the predicting region of *f*_1_(*z*), which suggests that its ncRNA fraction may be too small to be detected by the ncRNA/intron submodel, so the ncRNA fraction is estimated to be less than 5%. The preferred model suggests that the FANTOM3 stringent dataset with higher confidence for ncRNA annotation may contain a much higher fraction of genuine ncRNAs (47%) than the FANTOM3 dataset (18%).

We also predict the ncRNA fraction of the four computationally-identified datasets predicted by RNAz (the set1.P0.5, set1.P0.9, and set2.P0.5 datasets) and EvoFold (the EvoFold dataset) (Table 4). Our preferred model predicts that set1.P0.5 and set1.P0.9 contain 39% and 52% genuine ncRNAs, respectively. The other two datasets (RNAz set2. P0.5 and EvoFold) have high sequence randomness (i.e. small relative *z*-scores); they locate out of the predicting region of all three submodels. We thus propose that these two datasets contain less than 5% genuine ncRNAs. The four computationally-predicted datasets, although all based on comparative analysis, contain different ncRNA fractions.

The corresponding number of genuine ncRNAs in the six datasets also varies greatly: 6,125 for FANTOM3, 1,356 for FANTOM3 stringent, 35,754 for RNAz set1.P0.5, 18,712 for RNAz set1. P0.9, <1,020 for RNAz set2.P0.5, and <2,424 for EvoFold. Since some conserved RNA folds form clusters and share common RNA transcripts (Pedersen et al. 2006), the actual number of genuine ncRNAs in the computational predictions may be smaller than the above numbers.

### Thermodynamic analysis of the FANTOM3 putative ncRNAs

**Figure 6:**
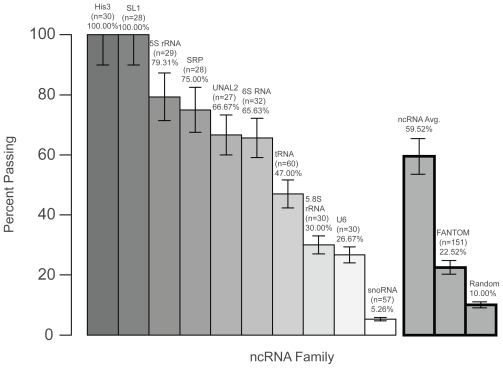
Thermodynamic analysis of selected sequences of the FANTOM3 putative ncRNA dataset (<400 nt) and ten known ncRNA families. The passing rate, tested sequence number and dataset name are shown above the passing rate bar.

In addition to developing the fraction model to assess putative ncRNAs, we also employ thermodynamic tests (involving free energy, melting temperature and energy landscape) to analyze 151 short (<400 nt) putative ncRNAs in FANTOM3; only short RNAs are used for this analysis to reduce errors in secondary folding algorithms. Among these short putative ncRNAs, 23% are thermodynamically stable (Fig. 6). We also analyze known ncRNA families: tRNA, 5S rRNA, 5.8S rRNA, 6S RNA, SRP RNA, SL1 RNA, U6 RNA, UnaL2, snoRNA, and His3. Except for the non-structural snoRNA family, the other nine ncRNA families have higher passing rates than the FANTOM3 subset. The average passing rate of the ten ncRNA families is 60%. This analysis shows that ncRNAs are more stable than both the random sequences and the FANTOM3 subset. It also suggests that 23% of short putative ncRNAs in FANTOM3 may have biological function, in agreement with the relative *z*-score analysis (18%).

### Systematic errors in the fraction model

**Figure 7:**
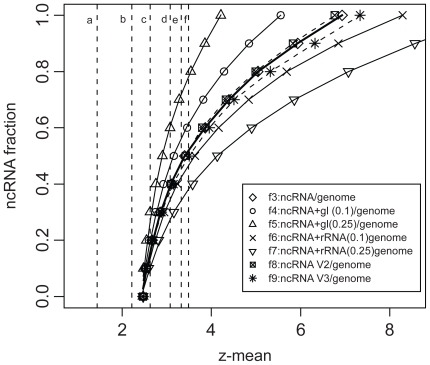
The six submodels used to simulate systematic errors in our fraction model. The *f*_4_ and *f*_5_ are constructed by 10% and 25% group I intron sequences in the ncRNA partition of the ncRNA/genome model, respectively; and the *f*_6_ and *f*_7_ are constructed by 10% and 25% rRNA sequences in the ncRNA partition, respectively. The submodels *f*_8_ and *f*_9_ are ncRNA/genome models using version 2 and version3 ncRNA reference dataset (Table 1), respectively. The mean relative *z*-score of the six test datasets is shown in dashed lines in the same order as Fig. 4.

Since the FANTOM3 data are assembled from diverse sources (tissues, experimental conditions, various laboratories, etc), the simplest and reasonable model for the FANTOM3 data is a collection of diverse RNA families similar to the composition of our training dataset. Still, systematic errors of the fraction model can arise due to possible differences in the composition of ncRNAs in training and test datasets. For example, the test datasets could be enriched with specifically low or high relative *z*-score ncRNAs. To simulate such datasets, we generate four biased fraction models containing 10% and 25% of group I intron (low relative *z*-score, data not shown) and rRNA (high relative *z*-score, data not shown) sequences in the ncRNA partition of the ncRNA/genome model, labeled *f*_4_, *f*_5_, *f*_6_ and *f*_7_, respectively. Figure 7 shows that the four biased models have different error ranges. For example, the predicted ncRNA fraction in the FANTOM3 stringent dataset increases from 47% to 56% and 72% for *f*_4_ and *f*_5_ (10% and 25% group I intron), but decreases to 42% and 34% for *f*_6_ and *f*_7_ (10% and 25% rRNA) (Supplementary Table S4). Thus, for datasets with enriched 10% random-like (e.g. group I intron) or structural ncRNAs (e.g. rRNA), the error would be ~10%. The two enriched 25% models are less likely due to the high diversity of our test data-sets (Supplementary Figs. S2–S4). These four biased models can partially simulate scenarios where the test datasets have compositions different from that of the training ncRNA dataset. Expected increase of experimentally characterized ncRNAs in the near future will help improve these fraction models.

We have also estimated the uncertainties arising from changing the composition of training dataset when some ncRNA families are removed. Table 1 shows two additional training datasets (versions 2 and 3) with low and no representation of rRNA, tRNA and spliceosome families. These datasets lead to only 0.5–1.5 standard deviations from the mean relative *z*-score of the original training dataset, implying errors of less than 5% in predicted ncRNA fraction.

## Discussion

We have proposed here a fraction model for assessing ncRNA content of sequence datasets based on a sensitive relative *z*-score for measuring the degree of sequence randomness. Our fraction model relies on the relative *z*-score to help distinguish the genome/intergene/intron cluster from the mRNA/ncRNA cluster. It assumes that the relative *z*-score can discriminate the ncRNA class and the genome/intergene/intron cluster (Fig. 3a), and that the training and test datasets have similar sequence conservation patterns (Figs. S1–S4). These are reasonable first-order approximations but only with much more data can these assumptions be validated.

Clearly, sequence randomness reflects some functional features. For example, ncRNAs likely contain recurrent motifs (e.g. GNRA, UNCG) underlying RNA’s modular architecture (Hendrix et al. 2005; Leontis et al. 2006). Of course, there are numerous other aspects that must be considered. Moreover, all statistical features analyzed here rely on currently available datasets. Though many more RNAs await discovery, the modularity of RNA and reliance on a few recurring motifs suggest that our approach is worth considering. It is interesting to recall that when the ribosome structure was solved, only a few *new* tertiary motifs emerged despite expectations to the contrary.

Other computational studies of ncRNA, most of which are based on comparative genomic analysis, such as QRNA (Rivas and Eddy, 2001), RNAz (Washietl et al. 2005b) and EvoFold (Pedersen et al. 2006), are limited by the requirement of high sequence conservation across species. However, many ncRNAs exhibit low sequence conservation (Pang et al. 2006). In contrast, the relative *z*-score assesses the randomness degree of any sequences whether conserved or not. Nevertheless, this approach is not applicable to single ncRNA sequences, because those sequences are three to four orders of magnitude shorter than the required length (2,097,152 nt) for reliable statistical analysis by the current approach. Though it may be possible to reduce the sequence length by changing the word size and then recalculating the mean value and standard deviation of Eq. 1, longer sequence lengths are more reliable for the monkey test application.

Our fraction model, if valid, predicts that less than 52% of putative ncRNAs predicted by FAN-TOM3 and computational approaches are functional. This is not consistent with the speculation that most of the putative ncRNAs are functional (Mattick and Makunin I.V. 2006) but agrees with other computational studies. For example, the EvoFold program predicted that 517 out of 48,479 conserved RNA folds are ncRNA candidates (Pedersen et al. 2006), which agrees with our prediction that less than 5% (<2,424 folds) are genuine ncRNAs. The RNAz program screened the dataset of FANTOM2 putative ncRNAs and only identified 781 out of more than 15,000 putative ncRNAs having conserved RNA secondary structures (Washietl et al. 2005a). This number is much less than our predicted number (6,125) in the FANTOM3 dataset partly because the relative *z*-score assesses both conserved and nonconserved sequences. In addition, false positives from computational predictions can contribute to over-counting of genuine ncRNAs. For example, the high false positive rate, 28.9% (p = 0.5), for the RNAz program suggests that only a part of predictions may be real ncRNAs.

Moreover, our fraction model assumes that putative ncRNAs contain genuine ncRNAs and background noise. At least three potential errors may be introduced into the model: (1) limited amount of training data for ncRNAs; (2) limited source of background noise; and (3) “contamination” of mRNAs in a tested dataset. For the first type of error, as the number and diversity of ncRNA families increase, the verity and precision of our fraction model can be assured and improved. The second type of error arises from the limited knowledge of transcriptome noise. Available experimental data indicate that over 60% of the mammalian genomes are transcribed (Carninci et al. 2005), but annotation is an ongoing process. Finally, as shown in our randomness analysis, the mRNA and ncRNA classes share a same region of randomness in the three-domain collection and Eukarya.

In conclusion, based on a first-level approximation, we suggest that fewer putative ncRNAs in datasets identified by experiments or computational approaches may exist. Clearly, our understanding of the general importance of ncRNAs in mammalian transcriptomes will advance as the number of genuine ncRNAs is better estimated rather than speculated. Our fraction model, if validated, might also be used to investigate ncRNA fraction of other putative ncRNA databases. The relative *z*-score may also help guide the detection of novel aptamers and ribozymes through design of sequence pools, an area of current work (Gevertz et al. 2005; Kim et al. 2007).

## Supplementary Material

**Table S1:** Selected genomic sequences in our randomness analysis of the three phylogenetic domains. Acc. No. denotes the accession number in GenBank.

Domain	Organism	Acc. No.	Length (nt)
	*Archaeoglobus fulgidus* DSM 4304	NC_000917	2,178,400
	*Halobacterium sp.* NRC-1	NC_002607	2,014,239
	*Methanococcus maripaludis* S2	NC_005791	1,661,137
	*Methanopyrus kandleri* AV19	NC_003551	1,694,969
	*Methanosarcina acetivorans* C2A	NC_003552	5,751,492
	*Methanosarcina mazei* Go1	NC_003901	4,096,345
	*Methanospirillum hungatei* JF-1	NC_007796	3,544,738
	*Methanothermobacter thermautotrophicus* str. Delta H	NC_000916	1,751,377
	*Natronomonas pharaonis* DSM 2160	NC_007426	2,595,2,097,152
Archaea	*Picrophilus torridus* DSM 9790	NC_005877	1,545,895
	*Pyrobaculum aerophilum* str. IM2	NC_003364	2,222,430
	*Pyrococcus abyssi* GE5	NC_000868	1,765,118
	*Pyrococcus furiosus* DSM 3638	NC_003413	1,908,256
	*Sulfolobus acidocaldarius* DSM 639	NC_007181	2,225,959
	*Sulfolobus solfataricus* P2	NC_002754	2,992,245
	*Sulfolobus tokodaii* str. 7	NC_003106	2,694,756
	*Thermococcus kodakarensis* KOD1	NC_006624	2,088,737
	*Thermoplasma acidophilum* DSM 1728	NC_002578	1,564,906
	*Thermoplasma volcanium* GSS1	NC_002689	1,584,804
	*Acinetobacter* sp. ADP1	NC_005966	3,598,621
	*Agrobacterium tumefaciens* str. C58	NC_003062	2,841,581
	*Azoarcus* sp. EbN1	NC_006513	4,296,230
	*Bacillus halodurans* C-125	NC_002570	4,202,352
	*Bordetella bronchiseptica* RB50	NC_002927	5,339,179
	*Caulobacter crescentus* CB15	NC_002696	4,016,947
	*Corynebacterium efficiens* YS-314	NC_004369	3,147,090
	*Chlorobium chlorochromatii* CaD3	NC_007514	2,572,079
	*Desulfitobacterium hafniense* Y51	NC_007907	5,727,534
Bacteria	*Enterococcus faecalis* V583	NC_004668	3,218,031
	*Escherichia coli* K12	NC_000913	4,639,675
	*Lactobacillus acidophilus* NCFM	NC_006814	1,993,564
	*Listeria monocytogenes* EGD-e	NC_003210	2,944,528
	*Neisseria gonorrhoeae* FA 1090	NC_002946	2,153,922
	*Prochlorococcus marinus* str. MIT 9312	NC_007577	1,709,204
	*Rhizobium etli* CFN 42	NC_007761	4,381,608
	*Rhodopirellula baltica* SH 1	NC_005027	7,145,576
	*Staphylococcus aureus* subsp. aureus MW2	NC_003923	2,820,462
	*Thermus thermophilus* HB27	NC_005835	1,894,877
	*Xanthomonas campestris* pv. Campestris str. 8004	NC_007086	5,148,708
	*Anopheles gambiae*	NW_045800.1	6,709,423
	*Arabidopsis thaliana*	NC_003071.3	19,705,359
	*Caenorhabditis elegans*	NC_003281.4	13,783,316
	*Danio rerio*	NW_634459.1	2,669,025
	*Danio rerio*	NW_634120.1	2,112,237
	*Drosophila melanogaster*	NT_033779.3	22,407,834
	*Drosophila melanogaster*	NC_004354.2	22,224,390
	*Homo sapiens*	NT_006316.15	22,487,426
Eukarya	*Homo sapiens*	NT_033903.7	14,395,596
	*Mus musculus*	NT_039305.5	37,613,096
	*Mus musculus*	NT_039474.5	26,734,816
	*Plasmodium falciparum*	NC_004316	2,271,477
	*Rattus norvegicus*	NW_047692.2	2,154,120
	*Rattus norvegicus*	NW_047511.1	2,865,177
	*Saccharomyces cerevisiae*	NC_001136.6	1,531,916
	*Saccharomyces cerevisiae*	NC_001147.4	1,091,287
	*Schizosaccharomyces pombe*	NC_003424.2	5,572,983

**Table S2:** The datasets used in the fraction model.

Class	Relative *z*	Sequences	Total length (nt)	Source
Genomic sequence (mouse)	2.47 (0.04)	100	1,232,506,963	GenBank (listed in Table S3)
Intergenic region (mouse)	2.36 (0.04)	14,615	840,729,376	GenBank (listed in Table S3)
Intron (mouse)	3.19 (0.10)	85,672	74,985,163	the Exon-Intron database
Non-coding RNA	6.93 (0.33)	7,698	2,451,312	RNAdb, Noncode, Rfam, European ribosomal RNA database

**Table S3:** 100 mouse genomic RefSeqs serve as sources for the mouse genome and intergene classes in our fraction model. Acce No. denotes the accession number in GenBank.

Acce No.	Acce No.	Acce No.	Acce No.
NT_039173	NT_039360	NT_039548	NT_039702
NT_039185	NT_039361	NT_039563	NT_039711
NT_039186	NT_039385	NT_039573	NT_039713
NT_039189	NT_039413	NT_039578	NT_078297
NT_039190	NT_039420	NT_039580	NT_078355
NT_039202	NT_039424	NT_039586	NT_078380
NT_039206	NT_039436	NT_039589	NT_078925
NT_039212	NT_039438	NT_039590	NT_080546
NT_039229	NT_039455	NT_039595	NT_081117
NT_039230	NT_039457	NT_039596	NT_082868
NT_039234	NT_039460	NT_039609	NT_095756
NT_039238	NT_039461	NT_039617	NT_108905
NT_039240	NT_039462	NT_039618	NT_108907
NT_039260	NT_039471	NT_039625	NT_109313
NT_039267	NT_039474	NT_039636	NT_109314
NT_039268	NT_039475	NT_039638	NT_109317
NT_039301	NT_039476	NT_039641	NT_109320
NT_039302	NT_039477	NT_039649	NT_110856
NT_039314	NT_039482	NT_039650	NT_111909
NT_039340	NT_039490	NT_039655	NT_111916
NT_039343	NT_039495	NT_039657	NT_161953
NT_039350	NT_039496	NT_039676	NT_162143
NT_039353	NT_039500	NT_039678	NT_162293
NT_039356	NT_039501	NT_039699	NT_162294
NT_039359	NT_039515	NT_039700	NT_163365

**Table S4:** The systematic errors caused by biased ncRNA training datasets. Four submodels *f*_4_–*f*_7_ are created to simulated biased training data using 10% and 25% group I intron and rRNA sequences, repectively. The submodel labels are same as Fig. 7.

Dataset	*f*_5_(*z*)	*f*_4_(*z*)	*f*_3_(*z*)	*f*_6_(*z*)	*f*_7_(*z*)
Fantom putative	31%	22%	18%	16%	12%
Fantom stringent	72%	56%	47%	42%	34%
RNAz set1.P0.5	60%	47%	39%	35%	28%
RNAz set1.P0.9	78%	61%	52%	47%	38%
RNAz set2.P0.5	<5%	<5%	<5%	<5%	<5%
EvoFold*	<5%	<5%	<5%	<5%	<5%

* Same dataset as described in Table 4.

## Figures and Tables

**Table 1:** The composition of the training ncRNA class and other two constructs.

Group	Training set	Version 2	Version 3	Source
Noncode ncRNA	4251	4251	4251	Noncode
RNAdb ncRNA	2204	2204	2204	RNAdb
rRNA	129	0	10	EID^a^
tRNA	78	0	10	Rfam
spliceosomal RNA	28	0	10	Rfam
Total sequence number	6690	6455	6485	
Relative *z*-score (mean)	6.93	6.77	7.33	
Relative *z*-score (std)	0.33	0.11	0.17	

a EID: European ribosomal RNA database.

**Table 2:** The six putative ncRNA datasets.

Dataset	Sequences	Total length (nt)	Ave length (nt)
FANTOM3 putative ncRNA	34,030	67,856,244	1,994
FANTOM3 stringent putative ncRNA	2,886	4,535,792	1,572
RNAz set1.P0.5	91,676	12,474,689	136
RNAz set1.P0.9	35,985	5,475,570	152
RNAz set2.P0.5	20,391	2,798,941	137
EvoFold	48,479	1,869,205	39

**Table 3:** Relative *z*-scores of the six nucleotide sequence classes in the three domains.

Class	Collection		Archaea		Bacteria		Eukarya	
	
	Range	Mean (std)	Range	Mean (std)	Range	Mean (std)	Range	Mean (std)
Genome	0.9–11.7	2.9 (2.2)	1.2–11.5	3.6 (2.5)	1.2–15.2	3.0 (2.3)	1.0–5.8	2.2 (1.0)
Intergene	1.3–2.2	1.6 (0.2)	1.2–1.3	1.2 (0.02)	4.1–5.1	4.6 (0.2)	1.4–2.2	1.7 (0.2)
Intron	1.6–2.0	1.8 (0.1)	/	/	/	/	1.6–2.0	1.8 (0.1)
mRNA	5.7–7.3	6.3 (0.2)	2.8–3.5	3.1 (0.2)	4.0–5.1	4.6 (0.3)	3.7–5.1	4.4 (0.3)
ncRNA	6.3–7.7	6.9 (0.3)	/	/	8.7–9.5	9.1 (0.1)	6.1–6.5	6.2 (0.1)
Repeat	24.5–230.8	58.0 (32.9)	24.5–230.8	58.0 (32.9)	24.5–230.8	58.0 (32.9)	24.5–230.8	58.0 (32.9)

**Table 4:** The ncRNA fractions predicted by the model. Relative *z*-scores are shown in mean values and standard deviations.

Dataset	Relative *z*	*f*_1_(*z*)		*f*_2_(*z*)		*f*_3_(*z*)	
		
		%	Sequence #	%	Sequence #	%	Sequence #
Fantom putative	2.63 (0.05)	<5%	<1,701	22%	7,487	18%	6,125
Fantom stringent	3.32 (0.06)	9.7%	280	50%	1,443	47%	1,356
RNAz set1. P0.5	3.08 (0.04)	<5%	<4,584	42%	38,503	39%	35,754
RNAz set1. P0.9	3.49 (0.04)	21%	7,557	53%	19,072	52%	18,712
RNAz set2. P0.5	2.21 (0.01)	<5%	<1,020	<5%	<1,020	<5%	<1,020
EvoFold^a^	~1.44 (0.02)	<5%	<2,424	<5%	<2,424	<5%	<2,424

a The relative *z*-score of the EvoFold dataset is estimated by concatenated sequences mixed with EvoFold predictions and known ncRNAs because the total length of the EvoFold dataset is shorter than the required length of the DNA test.
